# Glycated CD59: a novel biomarker for gestational diabetes

**DOI:** 10.1210/endrev/bnag010

**Published:** 2026-04-21

**Authors:** Michelle Toth Castillo, Shauntina Drayton Powell, Amy Biermann, Julia Torrey, Miguel Angel Luque-Fernandez, Jose Halperin

**Affiliations:** Department of Medicine, Brigham and Women's Hospital, Harvard Medical School, Boston, MA 02115, USA; Department of Medicine, Brigham and Women's Hospital, Harvard Medical School, Boston, MA 02115, USA; Department of Medicine, Brigham and Women's Hospital, Harvard Medical School, Boston, MA 02115, USA; Department of Medicine, Brigham and Women's Hospital, Harvard Medical School, Boston, MA 02115, USA; Department of Biostatistics, University of Granada, Granada, Spain; Department of Medicine, Brigham and Women's Hospital, Harvard Medical School, Boston, MA 02115, USA

**Keywords:** gestational diabetes mellitus, pregnancy-induced glucose intolerance, complement system, CD59 and glycated CD59, biomarkers

## Abstract

Gestational diabetes mellitus (GDM), defined as glucose intolerance that starts during pregnancy, represents a major public health challenge because it is a major cause of adverse maternal and fetal outcomes and presents a significantly high risk of diabetes, obesity, and cardiovascular disease for both the mother and the infant.

The diagnosis of GDM is currently made through oral glucose tolerance tests (OGTT); other markers of glycemic control have notably failed for GDM diagnosis. Since the treatment of GDM reduces the incidence of adverse pregnancy outcomes, screening for GDM with OGTTs in pregnancy weeks 24 to 28 is the standard of care in most nations worldwide. However, universal screening is difficult to achieve due, in part, to the fact that OGTTs are cumbersome and uncomfortable.

Thus, the importance of detecting glucose intolerance in pregnant women, the possibility of reducing with treatment the associated risks, the low sensitivity of glycemic markers in pregnancy, and the multiple problems associated with OGTTs highlight the significance of exploring alternative screening/diagnostic methods that are sensitive, accurate, and well-tolerated by patients.

In this review, we summarize our discovery, development, and clinical validation in six human studies of plasma glycated CD59 (pGCD59), the glucose-modified form of the key complement inhibitor CD59, as a biomarker for screening, diagnosis, and monitoring of GDM.

## Essential points

- GDM is a major cause of adverse pregnancy outcomes, and women with GDM are at an increased risk of postpartum development of diabetes
- Current guidelines from professional organizations recommend testing all pregnant women without prior diabetes with OGTTs at pregnancy weeks 24 to 28
- Women with GDM should also undergo OGTT 6 to 12 weeks postpartum to rule out conversion to clinical diabetes
- Because OGTTs are cumbersome and uncomfortable, there is a recognized need for alternative, simpler methods for screening/diagnosis of GDM
- Soluble glycated CD59 (GCD59), the glucose-modified form of the complement regulatory protein CD59, is an emerging diabetes biomarker
- Several human studies have evaluated the potential role of plasma GCD59 as a biomarker for GDM screening, diagnosis, and monitoring of GDM
- This review summarizes the results of those six human studies
- The conclusion is that pGCD59 represents a novel and promising biomarker offering improved screening and diagnostic capabilities for GDM, with significant implications for enhancing maternal and neonatal health outcomes

The prevalence of diabetes is reaching epidemic proportions, currently affecting over 500 million people worldwide. If it continues to increase at the current rate, diabetes may potentially affect ≈10% to 12% of the world population by 2035 (1). The magnitude of the global diabetes epidemic is highlighted by the fact that treatment of diabetes complications costs more than 10% of the total healthcare budgets worldwide (2).

In parallel with an epidemic rise in diabetes prevalence and compounded by a universal increase in obesity and sedentary lifestyle, there is a worldwide increase in the prevalence of gestational diabetes mellitus (GDM) (3, 4), defined as glucose intolerance that starts or is first recognized during pregnancy. GDM is the most common medical condition in pregnancy, affecting one in six pregnancies worldwide (5). Pregnancy-induced glucose intolerance is a major public health challenge because it is a major cause of adverse maternal and neonatal outcomes and heralds a mother and newborn pair at risk of diabetes, obesity, and cardiovascular disease (6-8).

The diagnosis of GDM is made through different versions of oral glucose tolerance tests (OGTT) that involve a glucose load followed by measurements of plasma glucose levels for the following 2 or 3 hours (9). Since the treatment of GDM reduces the incidence of adverse pregnancy outcomes (10, 11), screening for GDM with OGTTs in the late second trimester is the standard of care in most countries around the world (12-14). Universal screening is difficult to achieve because OGTTs are time-consuming, uncomfortable, and unreliable (15).

Importantly, OGTTs impose an arbitrary dichotomous definition of “normal” and “abnormal” on gestational glucose tolerance, failing to recognize the continuous association between maternal hyperglycemia and adverse pregnancy outcomes described in the proceedings of the seminal hyperglycemia and adverse pregnancy outcomes (HAPO) study (16). Indeed, empirical evidence accumulating over the last 30 years supports recognizing a population of pregnant women who fall in between the currently accepted “normal” and “abnormal” thresholds of glucose tolerance tests. This “intermediate” category of pregnancy-induced glucose tolerance is also associated with a considerably increased risk of fetal macrosomia (27.5% vs 9.9% in normal), preeclampsia, c-section delivery (40.0% vs 19.9%), preterm delivery, neonatal jaundice, and extended maternal and infant hospital stay (17-22). Clinical identification of women in this “intermediate” category is of critical clinical and public health significance because treatment substantially mitigates their increased risk of adverse pregnancy outcomes.

However, currently used markers of glycemic control have notably failed for GDM screening and diagnosis: (1) HbA1c is not recommended by practice guidelines (12-14) or used by obstetricians for GDM screening/diagnosis because of its poor sensitivity. Nonetheless, it is measured in the first trimester to rule out diabetes in pregnancy (DIP), which is diagnosed when values are higher than 6.5% (≥48 mmol/mol) (23). A large study involving 19 261 pregnant women concluded that HbA1c has limited value in the diagnosis of GDM (24). Notably, HbA1c's sensitivity is affected, in part, by the increased turnover rate of erythrocytes during pregnancy, compounded by the fact that in pregnant women with GDM, fasting glucose levels are usually not as high as in other diabetic conditions (25, 26). (2) Glycated albumin and fructosamine are also not recommended as GDM by practice guidelines. Several human studies demonstrated that these glycemic markers have very limited value as screening tests for GDM (27, 28) likely because of their low sensitivity to mild hyperglycemia. (3) Fasting plasma glucose (FPG) lacks sensitivity for GDM because the worsening insulin resistance in pregnancy is initially compensated by increased insulin production, which results in normal or close to normal FPG (26). In addition, the poor sensitivity of random plasma glucose precludes its use for GDM screening (26). (4) Continuous glucose monitoring (CGM) detects trends in blood glucose levels, thus reducing the risks of hypoglycemia episodes in insulin-treated individuals, but the complexity of its implementation prevents its use for GDM screening in large populations of pregnant women.

Several other biomarkers are currently being investigated for their potential role in the early diagnosis (reviewed in a previous study (29) and monitoring of GDM (Table 1 (30)). Adiponectin, an adipocyte-derived cytokine, has been shown to have anti-inflammatory and insulin-sensitizing properties, making it a promising candidate for identifying metabolic disturbances during pregnancy; however, its potential use as a sensitive and specific clinical test for the diagnosis of GDM has not been evaluated in large prospective human studies (30). C-reactive protein (CRP), a marker of systemic inflammation, has also been correlated with insulin resistance and may serve as an indicator of GDM risk, but it is not a marker of actual hyperglycemia (30).

**Table 1:** Biochemical and molecular biomarkers of GDM (30)

Biochemical biomarker	Main Source	Gestational age (weeks)
SHBG	Liver, placenta	1st-13th
Afamin	Liver, placenta	1st-12th
Ficolin-3	Liver, placenta	16th-18th
Fetuin-A	Liver, placenta	11th-14th
hs-CRP	Liver, pancreas	4th-6th/11th-14th
Visfatin	Adipose, placenta	11th-13th
Omentin-1	Adipose, placenta	12th-15th
Adiponectin	Adipose, breast	6th-32nd
Leptin	Adipose, breast	14th-20th/24th-28th
1,5-anhydroglucitol	Liver	—
Glycosylated fibronectin	Liver	—

Similarly, emerging research highlights the role of microRNAs in regulating metabolic pathways and insulin sensitivity, suggesting their potential utility as biomarkers for the early detection of GDM (31). However, these molecular biomarkers present several challenges, including significant variability in miRNA expression across various studies, non-standardized miRNA isolation procedures, measurement platforms, and normalization methods that still preclude their validation as valuable diagnostic tools for GDM (32). A recent study that examined changes in 1,5-anhydroglucitol (1,5AG) levels during gestation showed no association with glucose levels, concluding that 1,5AG is not useful in assessing glycemia in pregnant women (33). A comparative assessment of these alternative biomarkers with pGCD59 would be essential for defining their respective sensitivities and specificities and for determining the most effective approaches for early intervention.

Thus, the importance of detecting glucose intolerance in pregnant women, the possibility of reducing associated risks for the mother and newborn with treatment, and the low sensitivity of other glycemic markers in pregnancy highlight the significance of exploring alternative screening/diagnostic methods that are sensitive, accurate, and well-tolerated by patients (28, 34, 35).

In this review, we summarize our discovery, development, and clinical validation of plasma glycated CD59 (pGCD59), the glycated form of the key complement regulatory membrane protein CD59, as a novel biomarker for screening, diagnosis, and monitoring of GDM.

### The complement system and diabetes

The complement system is an effector of both adaptive and innate immunity. It is composed of more than 30 plasma and cell-membrane proteins that are synthesized by hepatocytes or locally in peripheral tissues (36-39).

**Figure 1:**
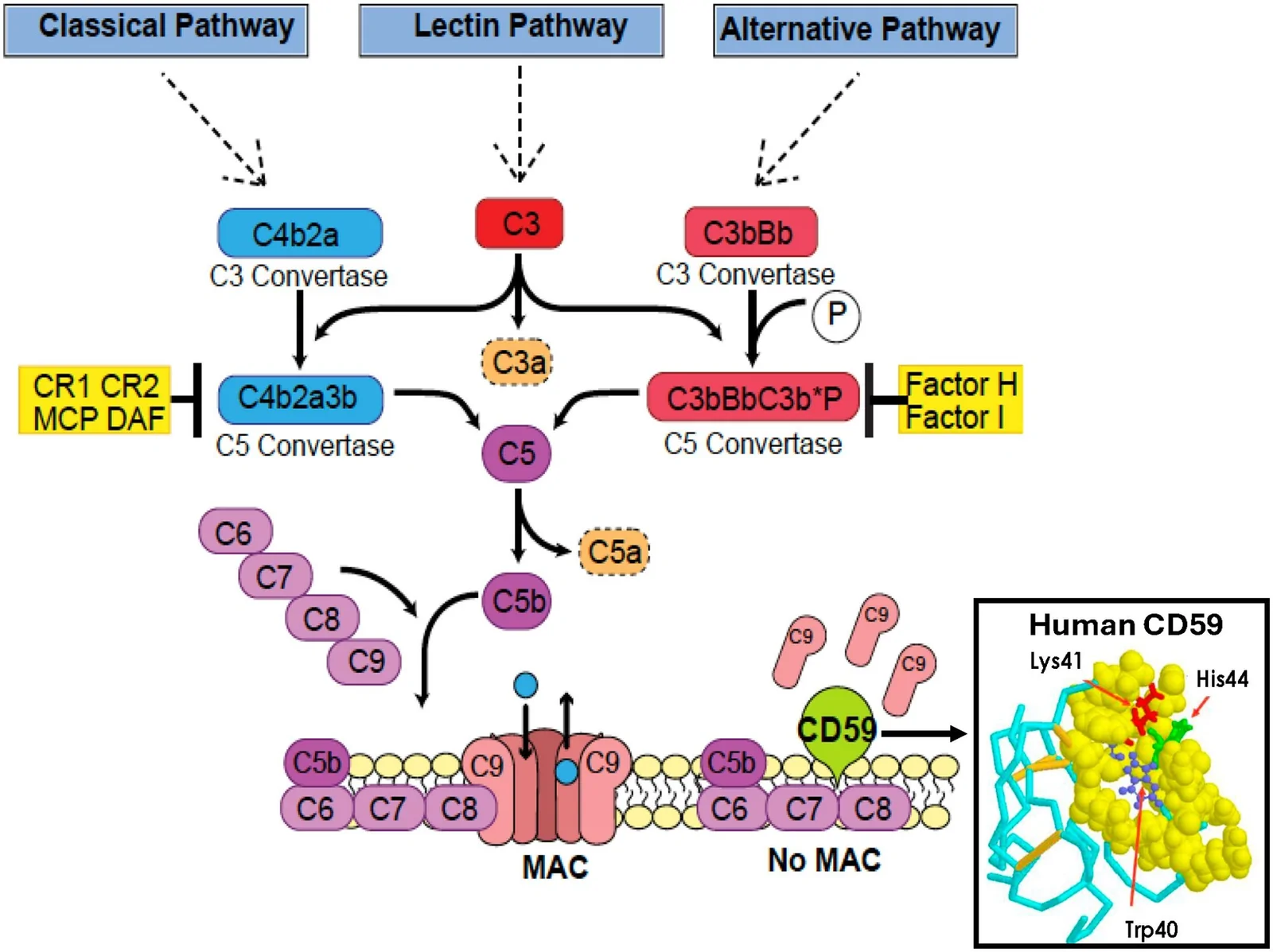
Convergence of the classical, lectin, and alternative complement activation pathways on the terminal complement cascade that leads to the formation of the MAC. The picture also depicts how CD59 inhibits the final assembly of the MAC by binding to the C5b678 complex, thus restricting C9 binding and polymerization (Adapted from Ghosh et al (43), Fig. 1). The *inset* depicts the NMR structure of human CD59, highlighting the glycation-prone motif formed by amino acid residues Lys41 (red) and His 44 (green), as well as the W40 residue that is essential for the MAC-inhibitory activity of CD59.

The complement system is activated through the interaction of its components via three enzymatic cascades known as the classical, the alternative, and the mannose-binding lectin (MBL) pathways (40-42). The three activation pathways converge at the level of C3 and C5; thereafter, the three complement pathways share a common reaction sequence through the late components C6, C7, C8, and C9, leading to the generation of the membrane attack complex (MAC), the main effector of complement-mediated tissue damage. The MAC is a circular polymer of C9 molecules that forms a transmembrane pore; insertion of the MAC pore allows the influx of salt and water, causing swelling and eventually lysis of the target cell (Fig. 1) (43).

Our laboratory has described how activation of the terminal complement cascade can also form transient MAC pores that open and close on the cell membrane without causing cell lysis (44, 45). Transient MAC pores can trigger an array of cellular responses that promote cell proliferation, inflammation, and thrombosis, as observed in all diseases in which focal complement activation is a distinctive pathological abnormality (46).

#### Complement regulatory proteins

The complement system is a potent threat to “self” cells because even small initiating stimuli may rapidly become magnified into a large and potentially harmful response. Given that uncontrolled complement activation can potentially have serious harmful effects, it is not surprising that several soluble and membrane-bound complement regulatory proteins, such as decay accelerating factor (DAF) and CD59 have evolved to restrict complement activation. Both DAF and CD59, ubiquitously expressed in mammalian cells, are linked to the external surface of the cell membrane by a glycosylphosphatidylinositol (GPI)-anchor. While DAF inhibits progression of the early complement cascade, CD59, an 18-20 kDa glycoprotein, is a specific inhibitor of MAC formation. The MAC-formation inhibitory activity of CD59 derives from its capacity to bind with high affinity to sites that are nascently exposed during assembly of the MAC complex on the target membrane, including the alpha-chain subunit of C8, and the domain conformed by amino acid residues 245 to 538 within the C9b component of complement; this, in turn, prevents the membrane insertion of C9 restricting C9 polymerization and MAC formation (Fig. 1) (43, 47-49).

Experimental and clinical evidence clearly indicates that CD59 is the most important complement regulator protecting “self” cells from unrestricted complement activation. Experimentally, DAF-deficient mice did not show a spontaneous hemolytic phenotype (50). In contrast, CD59 knockout mice present a strong phenotype characterized by hemolytic anemia with increased reticulocytes, free hemoglobin in plasma, hemoglobinuria with hemosiderinuria, and spontaneous platelet activation (51-53).

Clinically, an acquired absence of both CD59 and DAF on the surface of bone marrow-derived cells causes paroxysmal nocturnal hemoglobinuria (PNH), a complement-mediated hemolytic anemia associated with thrombosis (54), in which the biosynthesis of the glycosylphosphatidylinositol (GPI) anchor is deficient. However, subjects expressing the rare Inab erythrocytes phenotype have a hereditary DAF deficiency in erythrocytes that is not associated with clinically evident hemolytic disease (55). In contrast, a hereditary complete deficiency of CD59 due to a deletion in the CD59 gene caused severe PNH, as reported in (56, 57). Also, total absence of CD59 in five pediatric patients initially presenting with chronic inflammatory demyelinating polyradiculoneuropathy-like disease and chronic hemolysis eventually caused devastating recurrent brain ischemic infarctions and retinal disease (58, 59).

A peculiarity of human CD59 compared with CD59 from other species is the presence of a glycation-prone motif conformed by amino acid residues lysine 41 (L41) and histidine 44 (H44) within or very close to its active site that centers around the W40 amino acid residue. As we will detail below, the H44 residue of human CD59 is critical for the glycation-inactivation of human CD59 on L41 (60). The glycation-prone motif conformed by amino acid residues L41 and H44 in the human CD59 protein is shown in Fig. 1 inset. Whether other variants or mutations of the CD59 gene can impact its glycation, release into the circulation, and its detection by the current pGCD59 ELISA assay is not known at present and deserves further investigation.

#### Evidence for a role of complement in the pathogenesis of diabetes complications

A body of clinical and experimental evidence supports the link between the complement system, complement regulatory proteins, and the pathogenesis of diabetes complications (60-74).

#### Glycation-inactivation of CD59: a molecular link between complement and the complications of diabetes

Inactivation by glycation of CD59 is the most extensively studied and documented of the multiple mechanisms contributing to complement-mediated tissue damage in diabetes (43). In this review, we will summarize the experimental and clinical evidence that supports a critical role of GCD59 in the pathophysiology of diabetes complications.

Glycation of proteins on α- or ε-amino groups is a known mechanism of tissue damage in diabetes. Although many protein amino groups are amenable to glycation, only a few ε-amino groups are glycated to a significant extent. This is because effective acid-base catalysis required for ε-amino glycation occurs at “glycation motives” such as histidyl/lysyl residues, in which the histidyl is located in close proximity (≈5A°) to the ε-amino group of the lysyl residue, or at lysyl-lysyl pairs (75-79). For example, in the β-chain of hemoglobin, the lysyl residues K61, K65, and K66 are in very close proximity, but only the ε-amino group of K66, which is spatially close to the histidyl residue of H63, is preferentially glycated *in vivo* (75).

Analyzing the NMR structure of human CD59 (80), our group predicted that the protein contains a glycation-motif formed by amino acid residues K41 and H44, located in very close proximity to the protein active site (Fig. 1  *inset*). Based on the proximity of K41 to W40, an amino acid residue essential for CD59 function (81, 82), we further hypothesized that glycation could inhibit the activity of human CD59. Exposure of recombinant human CD59 to glucose helped demonstrate that the anti-MAC activity of human CD59 is indeed inhibited by glycation (60). Site-directed mutagenesis experiments confirmed that human CD59 is inhibited by glycation of the lysine 41 (K41) amino acid residue, a process that requires the close presence of the H44 residue (60).

Two lines of human experimental evidence seem to support the potential role of glycation-inactivation of CD59 in the pathogenesis of diabetes complications.

### Functional inactivation of CD59 in individuals with diabetes

If glycation inactivates CD59 *in vivo*, one would predict an increased sensitivity to MAC-mediated phenomena in cells/tissues carrying glycation-inactivated CD59. This prediction was confirmed in an *ex vivo* study using red blood cells (RBC) from donors with or without diabetes, in which we measured both the activity of CD59 in the RBC membranes and the RBC sensitivity to lysis by human MAC. The results showed that RBC from individuals with diabetes (HbA1c > 8%) were much more sensitive to complement-mediated lysis than RBC from individuals without diabetes (HbA1c < 5.5%). This increased sensitivity to lysis was associated with a significantly reduced activity of CD59, which is consistent with glycation-inactivation of CD59 (66). Glycation-inactivation of CD59 in RBC could explain some reversible hematological abnormalities suggestive of mild hemolytic anemia that have been reportedly observed in individuals with poorly controlled diabetes (83-85).

### GCD59 and MAC colocalize in the target organs of diabetic complications

Using a newly developed antibody that recognizes with high specificity the glycated from of CD59, we identified the presence of GCD59 in blood vessels, renal, and sural nerve biopsies from individuals with diabetes; no detectable amounts of GCD59 were found in biopsies from individuals without diabetes (66). Serial sections of the same renal and nerve biopsies showed co-localization of GCD59 with MAC deposits (66), as would be expected from the inactivation of CD59 function by glycation.

### Glycated CD59 as a diabetes biomarker

A soluble form of CD59 shed off from cell membranes by phospholipases is present in bodily human fluids, including blood, urine, and saliva (86-89). A highly specific and sensitive ELISA assay to measure the circulating levels of GCD59 was reportedly developed by Ghosh et al (90). This GCD59 ELISA is a ratio metric assay in which a capture monoclonal antibody binds “total CD59” (the glycated and non-glycated forms) and a detection antibody specifically binds to the CD59 molecules glycated on lysine 41 (90). Because the limited availability of GCD59 precludes its extensive use as an assay calibrator, the GCD59 ELISA is calibrated with a synthetic peptide, and the results are expressed in standard peptide units (SPU), as described in a previous study (90).

The concentration of CD59 in human plasma (≈10-12 ng/mL) is orders of magnitude higher than the concentration required to saturate the anti-CD59 specific capture antibody bound to the surface of the ELISA plate wells (89). For this reason, plasma samples for the assay are diluted ≈1000-fold (90).

In a human study conducted in 400 individuals to assess pGCD59 as a diabetes biomarker, levels of pGCD59 were 3-fold to 4-fold higher in individuals with type 2 diabetes, and independently associated with levels of HbA1c (43). In another study including 109 subjects with no prior diagnosis of diabetes, pGCD59 concentrations were strongly associated with higher 2-hour glucose levels following an OGTT, independently of other predictors of OGTT tests (43). Last, pGCD59 levels paralleled changes in glycemic control, as documented in an intensified insulin intervention study of individuals with poorly controlled diabetes; reductions in pGCD59 levels paralleled the reductions in average weekly glucose, both returning to almost baseline levels within ≈2 weeks. In contrast, HbA1c, fructosamine, and other markers of glycemic control did not change significantly over the initial 6 to 8 weeks of the study (43).

A recent study examined the relationship between plasma GCD59 levels and the presence of microvascular complications in 164 individuals with type 2 diabetes (T2DM), compared with 82 healthy controls, using a GCD59 assay kit manufactured in China. The authors concluded that pGCD59 is a promising biomarker for identifying T2DM patients who may be at increased risk for microvascular complications, independent of conventional glycemic markers (91).

The international multicenter continuous glucose monitoring in pregnant women with type 1 diabetes (CONCEPTT) study showed a strong association of GCD59 at 24 weeks with neonatal hypoglycemia and neonatal intensive care unit (NICU) admission (AUROC 0.72-0.73), performing better than HbA1c (AUROC 0.64-0.66) (92). The authors concluded that GCD59 could potentially play a role in the prediction of neonatal complications, particularly NICU admission and neonatal hypoglycemia. The predictive value of neonatal hypoglycemia was later confirmed in a sub-analysis of the vitamin D and lifestyle intervention (DALI) study, as reported in a previous study (93). Another study by Wang et al showed that levels of pGCD59 were significantly higher in a cohort of 113 women with GDM compared with 559 controls; however, the source of the assay reagents used in this study could not be verified (94).

### Complement, CD59, and pregnancy (normal and pathological)

Pregnancy constitutes a major challenge to the maternal immune system because it requires tolerance of fetal alloantigens encoded by paternal genes; failure of “tolerance” triggers fetal rejection, as it does in transplanted organs. The complement system plays a critical role in transplant rejection; similarly, excessive complement activation in the placenta places the fetus at risk for growth restriction or death (95). Therefore, it is not surprising that the fetus is protected from maternal immune responses by an array of mechanisms that include trophoblast expression of key complement regulatory proteins such as decay accelerator factor (DAF), membrane cofactor protein (MCP), and CD59. The key role played by complement and its regulators in pathological pregnancy is highlighted by experimental and clinical data showing that either immunologic maladaptation with activation of complement targeted to the placenta or decreased complement restriction (mutations of key complement regulators) contributes to the imbalance of angiogenic factors that is associated with placental dysfunction in preeclampsia (96, 97). Regarding pregnancy complications from diabetes, it is likely that glycation-inactivation of placental CD59 increases complement-mediated placental and fetal damage, contributing to the multiple pregnancy complications seen in women with GDM.

### GCD59 as a GDM biomarker

Several prospective and retrospective human studies have assessed the potential clinical utility of pGCD59 as a biomarker for GDM at critical time points during pregnancy and postpartum (98).

#### Detection of GDM at the standard of care screening window, weeks 24 to 28

A prospective human study reported in a previous study (99) was conducted in a cohort of 1000 pregnant women undergoing two-step screening for GDM showed that plasma levels of GCD59 at pregnancy weeks 24 to 28 were progressively higher across categories of both a 50-g/1 hour glucose tolerance test (normal glucose load test (GLT): 1 hour plasma glucose <140 mg/dL; failed GLT: 1 hour plasma glucose ≥140 mg/dL) and a 100 g/3 hours oral glucose tolerance test. The OGTT tests were adjudicated based on Coustan and Carpenter criteria (Normal: 0 or 1 abnormal value; GDM: 2+ abnormal values) (Table 2, from Reference 100).

**Table 2:** Median and Interquartile range of GCD59 in pregnant women with normal (controls) or abnormal (cases) glucose tolerance as determined by oral glucose load testing: GLT (1 hour Glucose Load Test) and OGTT (3-hours Oral Glucose Tolerance Test) at pregnancy weeks 24-28

Glucose load test category	Plasma GCD59 (SPUs) Median (IQR)	
.Normal GLT	0.33 (0.19)	*P* < .001
Failed GLT	2.79 (1.4)	
Failed GLT Normal OGTT	2.68 (1.31)	*P* of trend <.001
Failed GLT Abnormal OGTT (GDM)	3.23 (1.43)	

Reproduced from Ghosh et al (99), Table S2.

**Figure 2:**
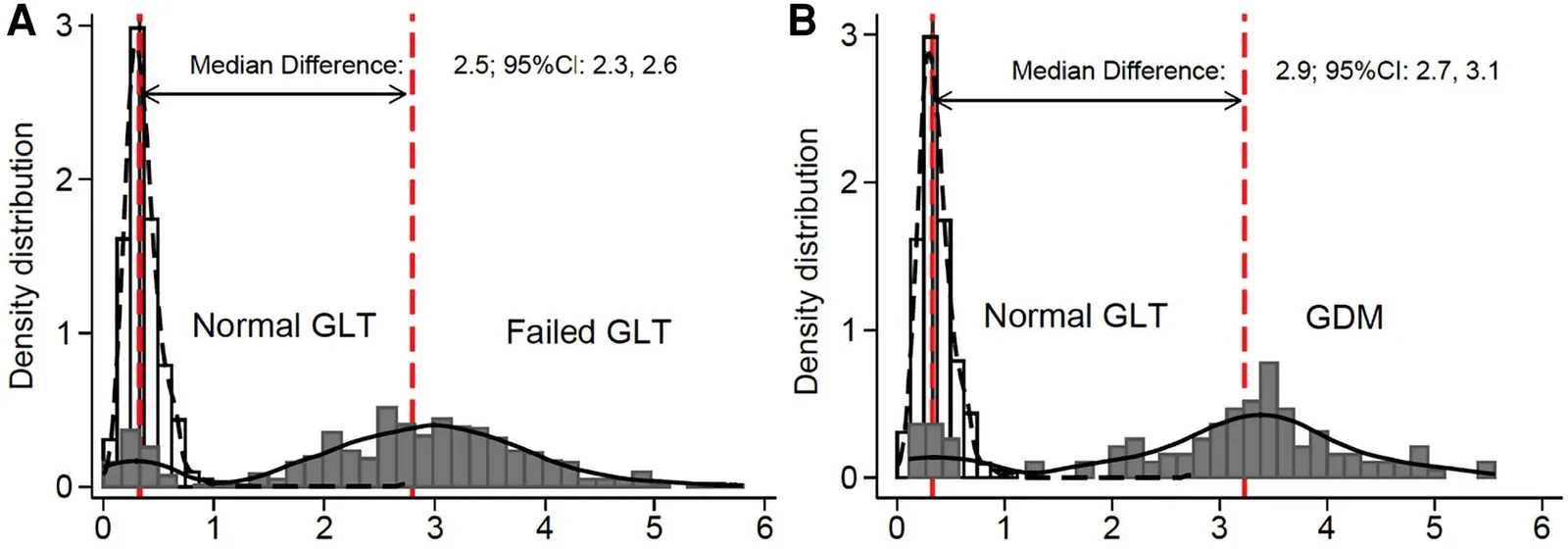
pGCD59 probability density functions between control vs failed GLT subjects (A) and control vs GDM subjects (B). The red dotted lines indicate the median pGCD59 values for the respective groups. The difference in median values between two groups and 95% CIs are shown in the figure. n = 1000 (Reproduced from Ghosh et al (99), Fig. 1A and 1B).

**Figure 3:**
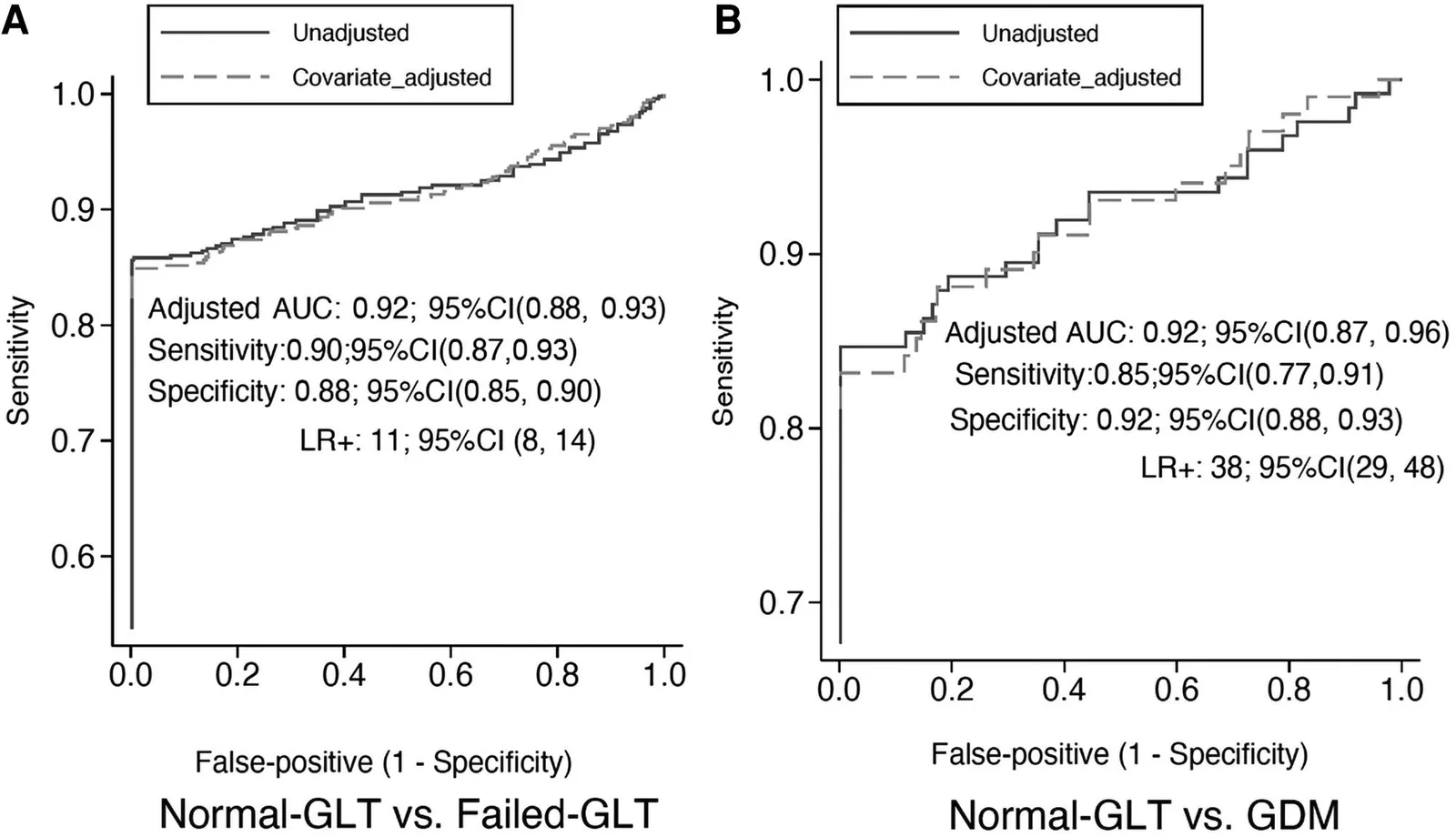
ROC curves by control vs failed GLT (A) and by control vs GDM subjects (B). ROC curves were computed and adjusted for maternal age, BMI, race/ethnicity, multiplicity, and gestational age at pGCD59 determination and history of diabetes. ***Dashed lines,*** ROC curves adjusted for maternal age, race/ethnicity, BMI, gestation week at pGCD59 determination, and a history of diabetes. The adjusted AUCs, sensitivity, and specificity with 95% CI. ***Solid lines***, unadjusted ROC curves. n = 1000 (Reproduced from Ghosh et al (99), Fig. 1C and 1D).

Furthermore, plasma levels of GCD59 discriminated with high sensitivity and specificity the normal GLT from the failed GLT populations (cases) as well as the normal GLT and the GDM cohorts (Fig. 2A and 2B). This was documented by areas under the receiver operating characteristic (ROC) curves (ROC AUC) of 0.92 (95% CI: 0.88, 0.93), even after adjustment for maternal age, body mass index (BMI), race, multiplicity, gestational age at pGCD59 measurement, and family history of diabetes (Fig. 3A and 3B). The positive and negative predictive values of pGCD59 to identify a woman who had a failed GLT were 99.3% (95% CI: 97.9, 99.8) and 87.5% (95% CI: 84.5, 90.1), respectively, and the positive likelihood ratio of having a failed GLT or GDM were +11 (95% CI: 8, 14) and +38 (95% CI: 29, 48).

Another prospective study of 387 pregnant women conducted at the Galway University Hospital in Ireland showed that pGCD59 levels were significantly higher in women with GDM compared to women with normal glucose tolerance (*P* = .003). In this study, however, mean values of pGCD59 in women with GDM were lower than in the study by Ghosh et al (99) (2.6 SPU vs 3.23), and the AUC for the overall population was 0.65 (100). This study was conducted using the 75 g-2 hours OGTT for GDM diagnosis, a methodology that tends to detect milder forms of GDM than the two-step approach used by Ghosh et al. As discussed by the authors, inclusion of milder forms of GDM and a much less ethnically diverse population are possible explanations for the lower AUC found in this study.

#### pGCD59 and large for gestational age (LGA) newborns

Plasma GCD59 was positively associated with the prevalence of large for gestational age (LGA) newborns, as determined by a birth-weight >90th percentile. Mothers who delivered an LGA newborn had a mean GCD59 level 10-fold higher than mothers who delivered a normal for gestational age newborn (99). Also, the prevalence of LGA newborns was approximately 10-fold higher in the highest than in the lowest quartile of plasma GCD59. Remarkably, 75% of the LGA newborns were born to mothers who had high levels of GCD59 but did not carry a formal diagnosis of GDM (99). A possible interpretation is that women diagnosed with GDM were treated and monitored during the pregnancy, reducing the risk of an adverse outcome. In contrast, women with pregnancy-induced glucose intolerance who did not meet formal criteria for GDM were not treated and monitored and therefore had a higher risk of birthing a hyperglycemia-induced LGA newborn.

#### GCD59 as a biomarker for early, first-trimester, detection of GDM

Women who develop GDM earlier in pregnancy are more likely to present maternal and fetal adverse outcomes, and their offspring are predisposed to diabetes and obesity (101-103). Indeed, women diagnosed with GDM before week 12 of gestation have similar pregnancy outcomes as women with pre-existing type 1 or type 2 diabetes (104). These reasons explain why expert panels in the United States and Europe recognized “early GDM diagnosis” as a major challenge and urged the conduct of studies to assess whether early diagnosis and treatment reduce adverse perinatal outcomes (4, 105). Those human studies aimed at critically assessing the effect of earlier diagnosis and treatment of GDM are impeded by a scarcity of biomarkers that are simpler and more reproducible than the glucose load tests, which are cumbersome and cannot be administered more than once in the course of routine prenatal care (15, 106). Since HbA1c and glycated albumin/fructosamine are not useful screening tests for GDM (27, 107, 108), there is a recognized need for alternate early pregnancy diagnostic markers of GDM.

**Figure 4:**
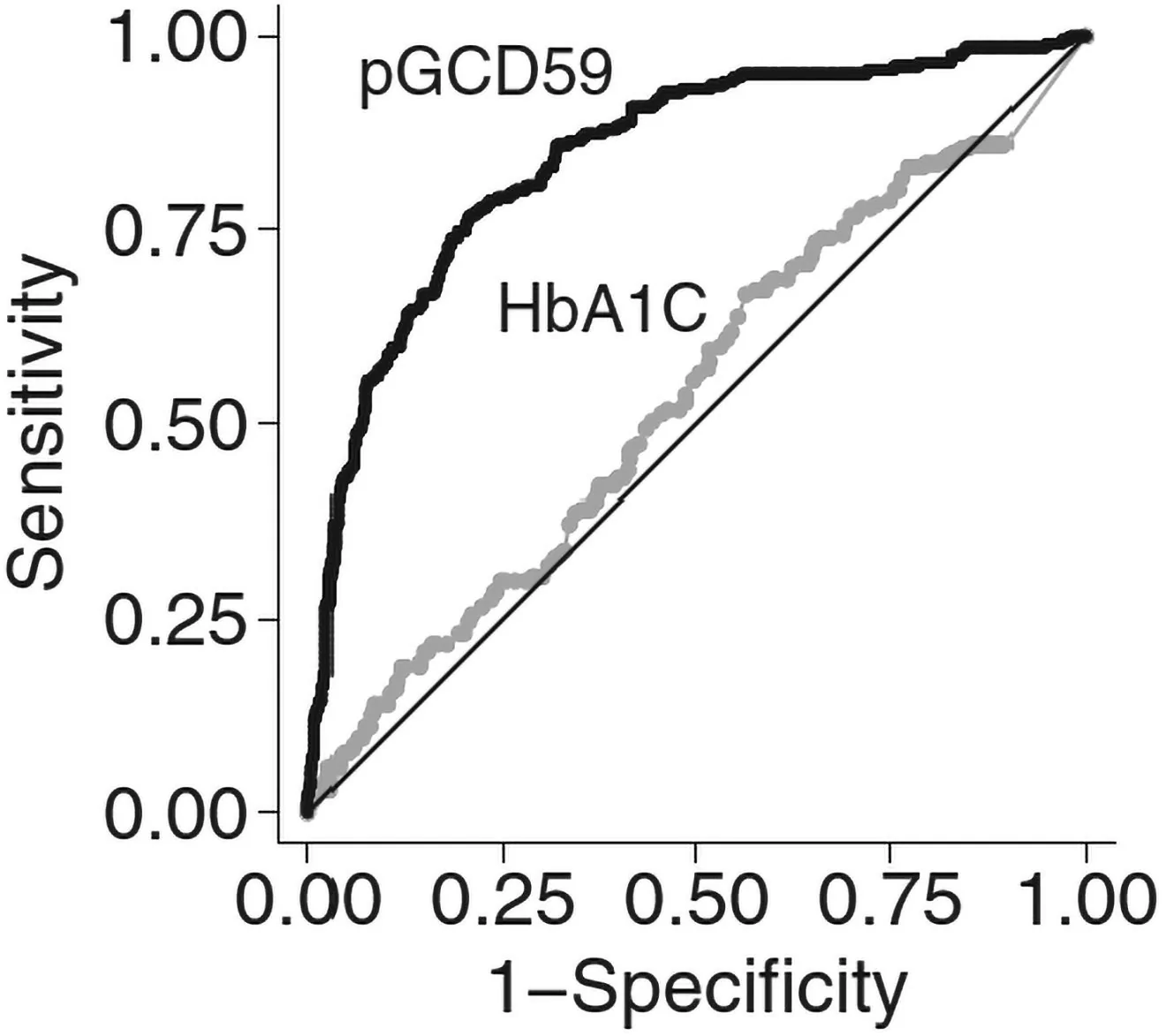
Comparison of pGCD59 and HbA1c ROC curves by non-GDM or GDM status diagnosed by OGTT testing at <20 weeks at pregnancy in the DALI study (reproduced from Ma et al (111), Fig. 2B).

To assess pGCD59 as a biomarker of early GDM and its association with delivering an LGA infant, blood levels of pGCD59 were measured in archival samples from 693 women undergoing a 75 g, 2-hour OGTT at <20-week gestation in the DALI study, a multicenter randomized controlled trial conducted across nine European countries (109, 110). The main analyses included 486 subjects who had normal glucose tolerance throughout the pregnancy, 207 who met criteria for GDM at <20 weeks, and 77 diagnosed with GDM at pregnancy weeks 24 to 28. The results showed that pGCD59 accurately identified GDM in early pregnancy with an AUC ROC curve of 0.86 (95% CI: 0.83-0.90), while HbA1c had no predictive value (AUC ROC of 0.54) (Fig. 4).

**Figure 5:**
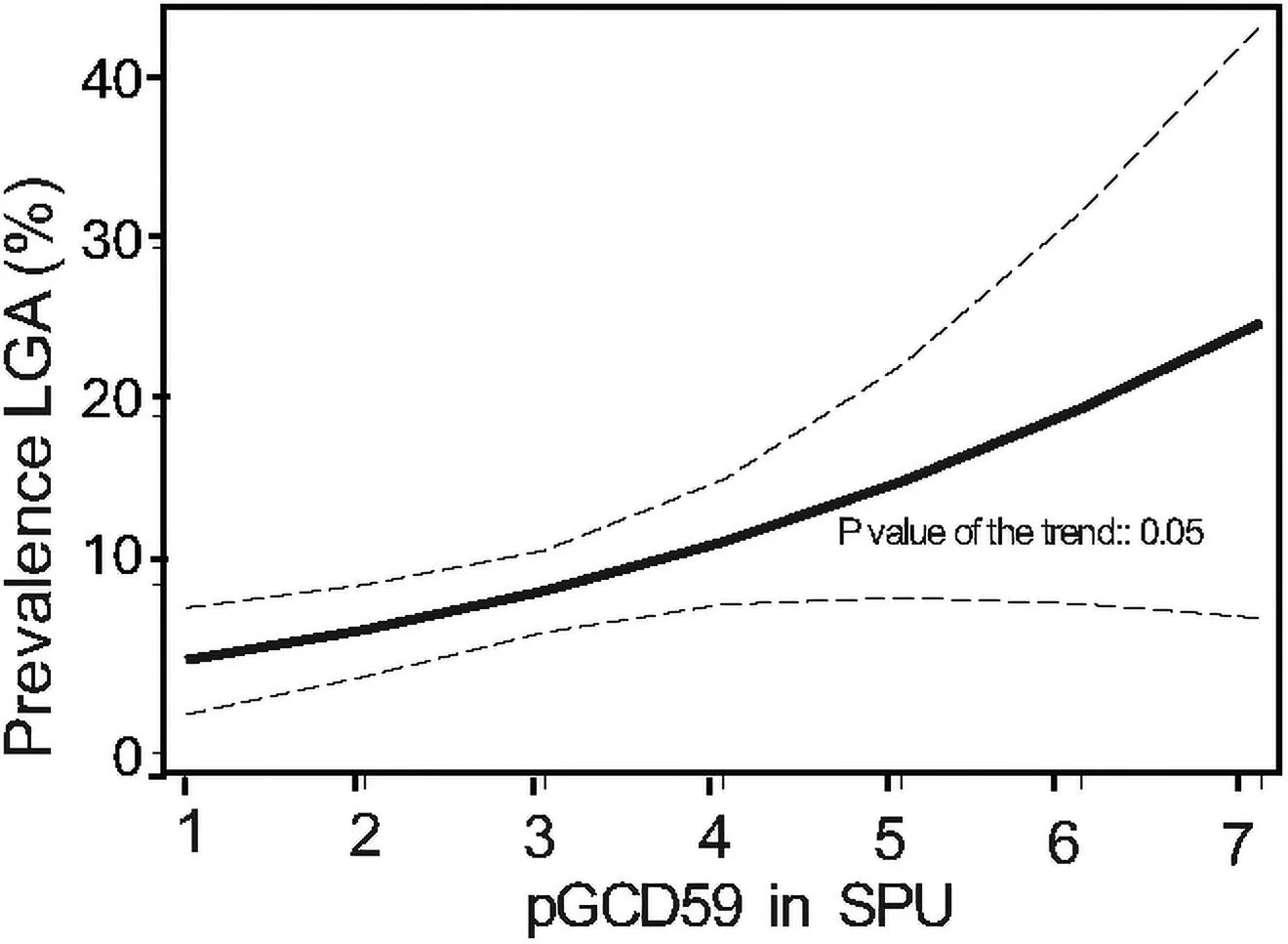
Trend of the association between maternal levels of pGCD59 at pregnancy week <20 and the prevalence of LGA in archival samples from 693 DALI study participants in which pGCD59 was measured (reproduced from Ma et al (112), Fig. 3).

Furthermore, higher maternal levels of pGCD59 were associated with the risk of delivering an LGA infant. Figure 5 shows the linear association of maternal levels of pGCD59 with the prevalence of LGA, adjusted for maternal age, ethnicity, gestation weeks, and BMI (trend test *P* = .05).

One unit increase in maternal pGCD59 level was associated with 36% increased odds of delivering an LGA infant (111).

Maternal levels of pGCD59 were also positively associated with the prevalence of neonatal hypoglycemia, where levels of pGCD59 at pregnancy weeks <20 identified the risk of neonatal hypoglycemia with a ROC AUC of 0.73 (95% CI: 0.63-0.83). Infants from mothers in the third tertile of pGCD59 values had approximately three times higher odds of neonatal hypoglycemia than those from mothers in the first tertile (93).

The utility of first-trimester pGCD59 for the detection of GDM was also assessed in a prospective study of 378 pregnant women in Ireland (113). In this study, pGCD59 was measured in blood samples drawn in the first trimester, but the diagnosis of GDM was made by 75 gr OGTT testing at weeks 24 to 28. The ability of first-trimester pGCD59 to predict the later development of GDM was assessed using ROC curves adjusted for maternal age, BMI, maternal ethnicity, parity, previous GDM, family history of diabetes mellitus, and week of gestation at the time of pGCD59 sampling. A sensitivity analysis by BMI subgroups showed that pGCD59 generated the highest AUC in the group with a BMI equal or above 35 kg/m^2^ but under 40 kg/m^2^ (AUC: 0.85, 95% CI: 0.70-0.98) and for those with a BMI equal or above 40 kg/m^2^ (AUC: 0.88, 95% CI: 0.63-0.99). The conclusion of this limited study was that pGCD59 in early pregnancy may be a good predictor of GDM in women with a high BMI.

#### Post-partum development of glucose intolerance

GDM imparts a 7-fold increased risk of subsequent development of T2DM compared with women without a history of GDM (112). Because HbA1c is not sensitive to detect glucose intolerance in early postpartum professional organizations such as the American Diabetes Association (ADA), American College of Obstetrics and Gynecology, and International Association of Diabetes and Pregnancy Study Groups recommend that women with GDM undergo OGTT testing 6 to 12 weeks postpartum (114). However, the cumbersome and time-consuming nature of OGTT testing makes compliance with this test very low, highlighting the need for alternative sensitive and easy-to-administer biomarkers that can be used postpartum to test women with GDM for potential conversion to T2DM.

The Belgian diabetes in pregnancy study (BEDIP-N), a prospective cohort study evaluating different screening strategies for GDM (115), assessed the accuracy of pGCD59 in predicting conversion to glucose intolerance diagnosed with an OGTT among women with GDM in the early postpartum. Postpartum levels of pGCD59 were a stronger predictor of glucose intolerance than HbA1c, as shown by an AUC ROC of 0.72 (95% CI 0.62-0.83) for pGCD59 and 0.50 (95% CI: 0.36-0.61) for HbA1c. A 0.5-unit increase in postpartum pGCD59 was associated with an odds ratio for glucose intolerance of 2.0 (95% CI 1.34-2.95, *P* < .001) (112).

These findings were confirmed in another prospective study of women with GDM in the Irish cohort. Postpartum pGCD59 measured at the time of OGTT identified women who developed glucose intolerance postpartum with a ROC AUC of 0.8. A main finding of this study was the 100% negative predictive value of a normal pGCD59, indicating that women who had GDM during pregnancy and a normal postpartum pGCD59 do not need to undergo glucose intolerance screening using the traditional 75 gr OGTT (116).

## Discussion

The human studies summarized in this review highlight the potential of pGCD59 as a novel and effective biomarker for the screening, diagnosis, and monitoring of GDM. The results consistently showed that pGCD59 is capable of identifying glucose intolerance during pregnancy with high sensitivity and specificity across different populations and stages of gestation, offering several advantages over currently available diagnostic measures.

Our findings reveal that elevated pGCD59 levels are associated with pregnancy-induced glucose intolerance as well as with the prevalence of adverse pregnancy outcomes traditionally linked to GDM, such as LGA newborns and neonatal hypoglycemia. This association supports the hypothesis that pGCD59 not only reflects the glycemic status during pregnancy but also provides a simple tool to stratify the risk of adverse outcomes, likely more precise than conventional OGTTs. The potential utility of pGCD59 as a biomarker in clinical settings is emphasized by its ability to identify women in the “intermediate” category of glucose intolerance who do not carry a formal diagnosis of GDM but are at higher risk of adverse pregnancy outcomes.

The work summarized in this review builds on previous literature that underscores the need for better markers to capture a spectrum of glucose intolerance that existing methods fail to address. Current tests for diagnosing GDM, including HbA1c, glycated albumin, and fructosamine, show limited sensitivity during pregnancy due to physiological changes affecting glucose metabolism. Indeed, mild forms of pregnancy-induced glucose intolerance are mostly reflected by post-prandial blood glucose peaks likely insufficient to glycate those other protein markers. The high sensitivity of pGCD59 to parallel the changes in glucose metabolism and provide a more direct marker of glucose dysregulation is likely related to the presence of a glycation-prone motif formed by amino acids lysine 41 and histidine 44; the imidazole ring of histidine 44 is close to lysine 41, providing acid-base catalysis for ε-amino glycation of lysine 41 (60).

## Conclusion

pGCD59 represents a novel and promising biomarker offering improved screening and diagnostic capabilities for GDM, with significant implications for enhancing maternal and neonatal health outcomes. The introduction of pGCD59 as a diagnostic tool in clinical practice could facilitate earlier and more accurate detection of GDM, enabling timely intervention to mitigate risks associated with maternal and fetal complications. Furthermore, the high negative predictive value of pGCD59 post-partum suggests its utility in screening women for the risk of developing T2DM after pregnancy, potentially reducing the need for cumbersome postnatal testing.

### Limitations and future research

While current findings on the diagnostic value pGCD59 in diagnosis of GDM are promising, further validation in larger cohorts and diverse populations is necessary to establish the generalizability of pGCD59 as a biomarker for GDM. Longitudinal studies could provide additional insights into how pGCD59 levels change throughout pregnancy and their relationship with long-term health outcomes for mothers and infants. Future research should also explore the reproducibility of the pGCD59 assay across different laboratories and testing platforms, the potential utility of the integration of pGCD59 screening in routine prenatal care, and its impact on healthcare costs and maternal outcomes.
